# Summary of the National Advisory Committee on Immunization (NACI) Seasonal Influenza Vaccine Statement for 2025–2026

**DOI:** 10.14745/ccdr.v51i09a01

**Published:** 2025-10-09

**Authors:** Katarina Gusic, Winnie Siu, Angela Sinilaite, Jesse Papenburg

**Affiliations:** 1 Centre for Immunization Surveillance and Programs, Public Health Agency of Canada, Ottawa, ON; 2 School of Epidemiology and Public Health, Faculty of Medicine, University of Ottawa, ON; 3 NACI Influenza Working Group Chair; 4 Division of Pediatric Infectious Diseases, Department of Pediatrics, Montréal Children’s Hospital, McGill University Health Centre, Montréal, QC; 5 Division of Microbiology, Department of Clinical Laboratory Medicine, OPTILAB Montréal, McGill University Health Centre, Montréal, QC; 6 Department of Epidemiology, Biostatistics, and Occupational Health, School of Population and Global Health, McGill University, Montréal, QC

**Keywords:** National Advisory Committee on Immunization, NACI, influenza, influenza vaccine, guidance

## Abstract

**Background:**

The National Advisory Committee on Immunization (NACI) reviews the evolving evidence on influenza immunization and provides annual recommendations regarding the use of seasonal influenza vaccines. The NACI *Statement on seasonal influenza vaccines for 2025–2026* updates the NACI recommendations from the previous year.

**Objective:**

To summarize the 2025–2026 NACI seasonal influenza vaccine recommendations and to highlight new and updated information.

**Methods:**

For the development of the *Statement on seasonal influenza vaccines for 2025–2026*, the NACI Influenza Working Group applied the NACI evidence-based process to assess available evidence and formulate recommendations. These recommendations were evaluated and approved by NACI based on the available evidence.

**Results:**

Key updates for the 2025–2026 influenza season include: 1) removal of the preferential recommendation for quadrivalent influenza vaccines in children; 2) reiteration of the safety of concurrent administration of seasonal influenza vaccines and other vaccines, including COVID-19, based on updated evidence; 3) new evidence on the protective effects of influenza vaccination on cardiovascular events; 4) updated language for Indigenous populations; and 5) addition of individuals at higher risk of avian influenza A(H5N1) exposure as a group for whom influenza vaccination is particularly important.

**Conclusion:**

NACI recommends that seasonal influenza vaccine should be offered annually to anyone six months of age and older who does not have a contraindication to the vaccine. Influenza vaccination is particularly important for people at high risk of influenza-related complications or hospitalization, people capable of transmitting influenza to those at high risk, and others as outlined in the Statement.

## Introduction

Canada experiences annual seasonal influenza epidemics, primarily in the late fall and winter. The burden of influenza-associated illness and death varies each year due to factors such as circulating virus type and affected populations ((1)). Globally, approximately 3 to 5 million cases of severe influenza illness and 290,000 to 650,000 deaths from influenza occur annually ((2)). In Canada, prior to the COVID-19 pandemic (i.e., 2010–2011 to 2018–2019 influenza seasons), influenza caused approximately 15,000 hospitalizations annually, more than any other seasonal respiratory virus ((3)). Vaccination remains the most effective form of protection against influenza and its complications.

The National Advisory Committee on Immunization (NACI) provides the Public Health Agency of Canada (PHAC) with annual recommendations on the use of authorized seasonal influenza vaccines, reflecting changes in epidemiology, immunization practices, and available products in Canada. The NACI Influenza Working Group (IWG) leads the annual update of the NACI statement on seasonal influenza vaccines, which involves a thorough review and evaluation of the literature, as well as discussion and debate at the scientific, clinical practice and population health levels. On April 30, 2025, PHAC released new guidance from NACI on the use of seasonal influenza vaccines for the 2025–2026 influenza season, based on current evidence and expert opinion. This article provides a concise summary of NACI’s recommendations and supporting information for the 2025–2026 influenza season, with emphasis on new or updated information since the 2024–2025 statement on seasonal influenza vaccines. For detailed information, refer to NACI’s *Statement on seasonal influenza vaccines for 2025–2026* ((4)).

## Methods

In preparation for the *Statement on seasonal influenza vaccines for 2025–2026*, the NACI IWG identified the need for evidence reviews on new topics, analyzed available evidence, and developed updated recommendations using NACI’s evidence-based process ((5)). Further details regarding the strength of NACI recommendations are available in **Table A1** in the **Appendix**. NACI’s peer-reviewed framework and evidence-informed tools (including the Ethics Integrated Filters, Equity Matrix, Feasibility Matrix, and Acceptability Matrix) were applied to help ensure that issues related to ethics, equity, feasibility and acceptability were systematically assessed and integrated into NACI guidance ((6)).

## Results

### Transition from quadrivalent to trivalent influenza vaccines

Previously, NACI recommended quadrivalent vaccines for children due to the higher burden of influenza B disease in this population and the extra protection conferred by the presence of both B/Victoria and B/Yamagata lineages in quadrivalent vaccines. As noted in the *Statement on seasonal influenza vaccine for 2024–2025* and its addendum, confirmed B/Yamagata virus infections have not been detected globally since March 2020, leading to expert groups, including PHAC, endorsing the exclusion of the B/Yamagata component from influenza vaccine formulations. This aligns with the World Health Organization (WHO) guidance for the 2024–2025 Northern Hemisphere season ((7–9)). Due to this changing epidemiology, NACI now has no preference between quadrivalent and trivalent vaccines and considers both formulations to be clinically safe and effective.

### Concurrent administration

NACI continues to recommend that all seasonal influenza vaccines (including live attenuated influenza vaccines [LAIV]) may be given at the same time as, or at any time before or after, administration of other vaccines (either live or non-live, including COVID-19 vaccines) for anyone six months of age and older. Evidence reviews on the concurrent administration of seasonal influenza vaccines (e.g., LAIV, inactivated influenza vaccines [IIV], recombinant influenza vaccines [RIV]) with other vaccines (e.g., COVID-19, respiratory syncytial virus, and pneumococcal) identified no safety, efficacy/effectiveness, or immunogenicity concerns. NACI will continue to monitor emerging evidence and update guidance as needed.

### Protective effects of influenza vaccination on cardiovascular events

Influenza infection has been associated with increased risk of cardiovascular events, including myocardial infarction, heart failure, and stroke ((10,11)). Following a literature review of existing systematic reviews and meta-analyses, NACI found supporting evidence for a protective effect of influenza vaccination against cardiovascular events in high-risk populations, such as those with underlying cardiovascular disease ((12)).

### Language pertaining to Indigenous peoples

In consultation with Indigenous immunization experts, NACI has updated its language on Indigenous peoples to specify “individuals in or from First Nations, Inuit, and Métis communities.” In addition, the rationale for including these individuals under the list of “Groups for whom influenza vaccination is particularly important” has also been updated to emphasize that the increased risk of severe influenza outcomes experienced by this group is due to multiple intersecting determinants of health, including social, environmental, and economic factors, rooted in historic and ongoing colonization and systemic racism (i.e., structural inequity).

### Guidance for people whose occupational or recreational activities increase their risk of exposure to avian influenza A(H5N1) viruses

Considering the ongoing outbreak of avian influenza A(H5N1) in humans and animals in countries such as the United States and Canada, NACI reiterates its recommendation that all individuals six months of age and older should receive a seasonal influenza vaccine. Although seasonal influenza vaccines do not protect against avian influenza infection, they may reduce the risk of seasonal and avian influenza A(H5N1) virus co-infection. Therefore, NACI has expanded its list of “Groups for whom influenza vaccination is particularly important” to include “people whose occupational or recreational activities increase their risk of exposure to avian influenza A(H5N1) viruses.” For preliminary guidance regarding the use of human vaccines against avian influenza, see the NACI rapid response on preliminary guidance on human vaccines against avian influenza as of December 2024 ((13)).

### Summary of NACI recommendations for the use of influenza vaccines for the 2025–2026 influenza season

**NACI recommends that any age-appropriate quadrivalent or trivalent influenza vaccine should be used for individuals six months of age and older who do not have contraindications or precautions.** Vaccination should be offered as a priority to people at high risk of influenza-related complications or hospitalization, people capable of transmitting influenza to those at high risk of complications, and others as indicated in **List 1**. Refer to **Table 1** for the recommended influenza vaccine products and **Table 2** for the recommended dose and administration route for each age group.

**Table 1:** Recommendations on choice of influenza vaccine type for individual and public health program-level decision making by age group

**People at high risk of influenza-related complications or hospitalization:** • All children 6 to 59 months of age • Adults and children with the following chronic health conditions^a^: o Cardiac or pulmonary disorders (including bronchopulmonary dysplasia, cystic fibrosis, and asthma) o Diabetes mellitus and other metabolic diseases o Cancer, immune compromising conditions (due to underlying disease, therapy, or both, such as solid organ transplant or hematopoietic stem cell transplant recipients) o Renal disease o Anemia or hemoglobinopathy o Neurologic or neurodevelopmental conditions (includes neuromuscular, neurovascular, neurodegenerative, neurodevelopmental conditions, and seizure disorders [and, for children, includes febrile seizures and isolated developmental delay], but excludes migraines and psychiatric conditions without neurological conditions) o Class 3 obesity (defined as body mass index of 40 kg/m^2^ and over) o Children 6 months to 18 years of age undergoing long-term treatment with acetylsalicylic acid, because of the potential increase of Reye’s syndrome associated with influenza • All pregnant women and pregnant individuals • All individuals of any age who are residents of nursing homes and other chronic care facilities • Adults 65 years of age and older • Individuals in or from First Nations, Inuit, or Métis communities as a result of intersecting determinants of health rooted in historic and ongoing colonization and systemic racism **People capable of transmitting influenza to those at high risk:** • Healthcare and other care providers in facilities and community settings who, through their activities, are capable of transmitting influenza to those at high risk • Household contacts, both adults and children, of individuals at high risk, whether or not the individual at high risk has been vaccinated: o Household contacts of individuals at high risk o Household contacts of infants less than 6 months of age, as these infants are at high risk but cannot receive influenza vaccine o Members of a household expecting a newborn during the influenza season • Those providing regular childcare to children 0 to 59 months of age, whether in or out of the home • Those who provide services within closed or relatively closed settings to people at high risk (e.g., crew on a cruise ship) **Others:** • People who provide essential community services • People whose occupational or recreational activities increase their risk of exposure to avian influenza A(H5N1)

**Table 2:** Recommended dose and route of administration, by age, for influenza vaccine types authorized for the 2025–2026 influenza season^a^

Age group	Influenza vaccine type (Route of administration)						Number of doses required
	IIV-SD^b^ (IM)	IIV-cc^c^ (IM)	IIV-Adj^d^ (IM)	IIV-HD^e^ (IM)	RIV^f^ (IM)	LAIV^g^ (Intranasal)	
6–23 months^h^	0.5 mL^i^	0.5 mL	0.25 mL	N/A	N/A	N/A	1 or 2^j^
2–8 years	0.5 mL	0.5 mL	N/A	N/A	N/A	0.2 mL (0.1 mL per nostril)	1 or 2^j^
9–17 years	0.5 mL	0.5 mL	N/A	N/A	N/A	0.2 mL (0.1 mL per nostril)	1
18–59 years	0.5 mL	0.5 mL	N/A	N/A	0.5 mL	0.2 mL (0.1 mL per nostril)	1
60–64 years	0.5 mL	0.5 mL	N/A	N/A	0.5 mL	N/A	1
65 years and older	0.5 mL	0.5 mL	0.5 mL	0.7 mL	0.5 mL	N/A	1

Abbreviations: IIV-Adj, adjuvanted inactivated influenza vaccine; IIV-cc, mammalian cell culture based inactivated influenza vaccine; IIV-HD, high-dose inactivated influenza vaccine; IIV-SD, standard-dose inactivated influenza vaccine; IM, intramuscular; LAIV, live attenuated influenza vaccine; N/A, not applicable; RIV, recombinant influenza vaccine

^a^ Given the global transition to trivalent influenza vaccines, the availability of various influenza vaccine preparations in Canada is evolving. Should the availability of a specific vaccine change (i.e., be made available or unavailable) after the release of this statement and prior to the 2025–2026 influenza vaccine season, NACI will communicate relevant information regarding the new vaccine preparations, if required

^b^ Afluria® Tetra (five years and older), Flulaval® Tetra (six months and older), Fluzone® Quadrivalent (six months and older), Influvac® Tetra (six months and older)

^c^ Flucelvax® Quad (six months and older)

^d^ Fluad Pediatric^TM^ (6 to 23 months) or Fluad® (65 years and older)

^e^ Fluzone® High-Dose Quadrivalent (65 years and older)

^f^ Supemtek® (18 years and older)

^g^ FluMist® Quadrivalent (2 to 59 years)

^h^ There is insufficient evidence for recommending vaccination with Influvac® Tetra (IIV4-SD) in children younger than three years of age

^i^ Evidence suggests moderate improvement in antibody response in infants, without an increase in reactogenicity, with the use of full vaccine doses (0.5 mL) for unadjuvanted inactivated influenza vaccines. This moderate improvement in antibody response without an increase in reactogenicity is the basis for the full dose recommendation for unadjuvanted inactivated vaccine for all ages. For more information, refer to the *Statement on Seasonal Influenza Vaccine for 2011–2012* ((17))

^j^ Children six months to less than nine years of age receiving seasonal influenza vaccine for the first time in their life should be given two doses of influenza vaccine, with a minimum interval of four weeks between doses. Children six months to less than nine years of age who have been vaccinated with one or more doses of seasonal influenza vaccine in the past should receive one dose of influenza vaccine per season thereafter

**List 1: Groups for whom influenza vaccination is particularly important**

^a^ Refer to immunization of persons with chronic diseases ((14)) and immunization of immunocompromised persons ((15)) in Part 3 of the *Canadian Immunization Guide* for additional information about vaccination of people with chronic diseases

**Table:** 

Recipient by age group	Vaccine types authorized and available for use	Recommendations on choice of influenza vaccine
6–23 months	IIV-Adj IIV-SD IIV-cc	• Any age-appropriate quadrivalent or trivalent influenza vaccine should be used for infants and young children who do not have contraindications or precautions, noting the following considerations: o Currently, there is insufficient evidence for recommending vaccination with Influvac® Tetra (IIV4-SD) in children younger than 3 years of age
2–17 years^a^	IIV-SD IIV-cc LAIV	• Any age-appropriate quadrivalent or trivalent influenza vaccine should be used for children and adolescents who do not have contraindications or precautions (see text below applicable to LAIV), including those with chronic health conditions, noting the following considerations and exceptions: o Currently, there is insufficient evidence for recommending vaccination with Influvac® Tetra (IIV4-SD) in children younger than 3 years of age • LAIV may be given to children with: o Stable, non-severe asthma o Cystic fibrosis who are not being treated with immunosuppressive drugs (e.g., prolonged systemic corticosteroids) o Stable HIV infection, i.e., if the child is currently being treated with ART for at least 4 months and has adequate immune function • LAIV should not be used in children or adolescents for whom it is contraindicated or for whom there are warnings and precautions, such as those with: o Severe asthma (defined as currently on oral or high-dose inhaled glucocorticosteroids) or active wheezing o Medically attended wheezing in the 7 days prior to vaccination o Current receipt of long-term aspirin or aspirin-containing therapy o Immune compromising conditions, with the exception of stable HIV infection, i.e., if the child is currently being treated with ART for at least 4 months and has adequate immune function o Pregnancy: - In pregnancy, IIV-SD or IIV-cc should be used instead
18–59 years	IIV-SD IIV-cc RIV LAIV	• Any of the available influenza vaccines authorized for this age group should be used for adults 18 to 59 years of age without contraindications or precautions, noting the following considerations and exceptions: o There is some evidence that IIV may provide better efficacy than LAIV in healthy adults • LAIV is not recommended for: o Pregnant women and pregnant individuals - In pregnancy, IIV-SD, IIV-cc, or RIV should be used instead o Adults with any of the chronic health conditions identified in List 1, including immune compromising conditions o Healthcare workers (HCWs)
60–64 years	IIV-SD IIV-cc RIV	Any of the available influenza vaccines authorized for this age group should be used for adults 60 to 64 years of age without contraindications or precautions.
65 years and older^b^	IIV-Adj IIV-SD IIV-HD IIV-cc RIV	IIV-HD, IIV-Adj, or RIV should preferentially be offered, when available, over other influenza vaccines for adults 65 years of age and older. If a preferred product is not available, any of the available influenza vaccines authorized for this age group should be used.

Abbreviations: ART, antiretroviral therapy; IIV, inactivated influenza vaccine; IIV-Adj, adjuvanted inactivated influenza vaccine; IIV-SD, standard-dose inactivated influenza vaccine; IIV-cc, mammalian cell–culture-based inactivated influenza vaccine; IIV-HD, high-dose inactivated influenza vaccine; IIV4-SD, standard-dose quadrivalent inactivated influenza vaccine; LAIV, live attenuated influenza vaccine; RIV, recombinant influenza vaccine

^a^ Refer to Table 3 in the Statement on seasonal influenza vaccines for 2025–2026 for a summary of vaccine characteristics of LAIV compared with IIV in children 2 to 17 years of age

^b^ Refer to the NACI supplemental statement on influenza vaccination in adults 65 years of age and older ((16)) for rationale, supporting evidence appraisal, and additional details on the evidence reviews that were conducted to support this recommendation

## Conclusion

NACI continues to recommend annual influenza vaccination for all individuals aged six months and older, noting product-specific age indications and contraindications. Influenza vaccination is particularly important for people at high risk of influenza-related complications or hospitalization; people capable of transmitting influenza to those at high risk; people who provide essential community services; and people whose occupational or recreational activities increase their risk of exposure to avian influenza A viruses (e.g., H5N1). Regarding updates for the 2025–2026 influenza season, NACI: 1) recommends that any age-appropriate quadrivalent or trivalent influenza vaccine should be used for individuals aged six months and older without contraindications or precautions; 2) continues to recommend that influenza vaccines may be given on the same day or at any time before or after other vaccines, including COVID-19 vaccines; and 3) lists individuals whose occupational or recreational activities increase their risk of exposure to avian influenza A(H5N1) viruses as a group for whom influenza vaccination is particularly important.

## Appendix

**Table A1:** Strength of the National Advisory Committee on Immunization recommendations

Strength of NACI recommendations (based on factors not isolated to strength of evidence, e.g., public health need)	Strong	Discretionary
Wording	“should/should not be offered”	“may be considered”
Rationale	Known/anticipated advantages outweigh known/anticipated disadvantages (“should”) OR known/anticipated disadvantages outweigh known/anticipated advantages (“should not”)	Known/anticipated advantages closely balanced with known/anticipated disadvantages OR uncertainty in the evidence of advantages and disadvantages exists
Implication	A strong recommendation applies to most populations/individuals and should be followed unless a clear and compelling rationale for an alternative approach is present	A discretionary recommendation may be considered for some populations/individuals in some circumstances Alternative approaches may be reasonable

Abbreviation: NACI, National Advisory Committee on Immunization
